# Framing the Drivers of Antimicrobial Resistance in Tanzania

**DOI:** 10.3390/antibiotics10080991

**Published:** 2021-08-16

**Authors:** Anna Durrance-Bagale, Anne-Sophie Jung, Gasto Frumence, Leonard Mboera, Stephen E. Mshana, Calvin Sindato, Taane G. Clark, Mecky Matee, Helena Legido-Quigley

**Affiliations:** 1 London School of Hygiene and Tropical Medicine, London WC1H 9SH, UK; anne-sophie.jung@lshtm.ac.uk (A.-S.J.); Taane.Clark@lshtm.ac.uk (T.G.C.); Helena.Legido-Quigley@lshtm.ac.uk (H.L.-Q.); 2 Muhimbili University of Health and Allied Sciences, Dar es Salaam 11103, Tanzania; frumencegasto@yahoo.co.uk (G.F.); mateemecky@yahoo.com (M.M.); 3 SACIDS Foundation for One Health, Sokoine University of Agriculture, Morogoro 65125, Tanzania; lmboera@gmail.com (L.M.); stephen72mshana@gmail.com (S.E.M.); csindato@gmail.com (C.S.); 4 Catholic University of Health and Allied Science, Mwanza 33109, Tanzania; 5 Tabora Research Centre, National Institute for Medical Research, Tabora 45026, Tanzania

**Keywords:** antimicrobial resistance, Tanzania, drivers, qualitative analysis, policy

## Abstract

Despite global awareness of the key factors surrounding antimicrobial resistance (AMR), designing and implementing policies to address the critical issues around the drivers of AMR remains complex to put into practice. We identified prevalent narratives and framing used by epistemological communities involved in the response to AMR in Tanzania, interrogated how this framing may inform policymaking, and identified interventions that could be tailored to the groups believed responsible for AMR. We interviewed 114 key informants from three districts and analysed transcripts line by line. Our results suggest that many different groups help drive the spread of AMR in Tanzania and need to be involved in any effective response. Human health is currently perceived as driving the response, while other domains lag behind in their efforts. For AMR programmes to be successful, all sectors need to be involved, including civil society groups, community representatives, and those working in communities (e.g., primary care physicians). However, current plans and programmes largely fail to include these viewpoints. The perceived presence of political will in Tanzania is a significant step towards such a response. Any strategies to tackle AMR need to be tailored to the context-specific realities, taking into account constraints, beliefs, and power dynamics within countries.

## 1. Introduction

While global awareness of the key drivers involved in antimicrobial resistance (AMR) exists, designing and implementing policies to address these critical issues is complex and must take into account many different factors. The situation is complicated by many factors, including political will, poverty, lack of understanding in communities and by experts, siloing of the various fields that need to take responsibility for both policy-setting and implementation (human, animal, and environmental health), and conflicts of interest. Preventing over- and misuse of antimicrobials needs to be balanced against ensuring equitable access, so that those who need them get them appropriately [1]. A further issue is that of counterfeit or substandard drugs, which are often widely available in markets and pharmacies in low- and middle-income countries (LMIC) [2]. Numerous stakeholders from the public, private, and civic sectors are involved in both perpetuating and potentially solving the problem, and lack of cooperation among these stakeholders may complicate AMR even further. For example, clinicians may be influenced to prescribe unnecessary antibiotics by pharmaceutical companies, and this type of conflict of interest needs to be strictly regulated [3]. Untangling these conflicts and setting priorities is not simple.

In February 2016, Tanzania was the seventh country to undergo a Joint External Evaluation (JEE) to examine preparedness to address public health threats, including AMR [4]. Tanzania had the lowest score (1) on three of the four AMR indicators (AMR detection, surveillance of infections caused by antimicrobial-resistant pathogens, and antimicrobial stewardship activities) and a score of 3 on the fourth (healthcare-associated infection prevention and control programmes). A score of 1 suggests no capacity, while a score of 3 reflects developed capacity. A score of 4 is the minimum desired target, indicating functionality and sustainability [5]. According to the JEE, many antimicrobials are used inappropriately in the animal and human health sectors in Tanzania, and no system is in place to collect data on the prevalence of resistance in common pathogens [4].

The Tanzanian National Action Plan (NAP) on AMR 2017–2022 was finalised in April 2017 and details actions to be taken to address issues around AMR [6]. The main points covered are the need to increase public awareness and promote behaviour change, strengthen surveillance and research, improve infection prevention and control systems, and improve the regulatory framework around antimicrobial agents to promote better stewardship of these drugs.

Effective implementation of the NAP in Tanzania is complicated by many familiar factors including (i) over-the-counter sales of antibiotics without a prescription [7], (ii) lack of effective regulation of antimicrobial use in animals and humans [8], (iii) over- and misuse of antimicrobials in livestock [9,10,11], (iv) poor surveillance [12], (v) self-medication [13], and (vi) the fundamental issue of lack of awareness of the existence of the Tanzanian NAP for AMR [12]. The key is to ensure appropriate access to antimicrobials while reducing overall use and promoting awareness in One Health practitioners and communities alike. One reported strength in Tanzania is the political commitment to addressing AMR, supported by leadership from the national government, which is helping to drive the coordination of effective interventions and efficient allocation of resources in the country [14].

Frames ‘define problems […] diagnose causes […] make moral judgments […] and suggest remedies’ [15]. Framing is used to suggest that some elements of a ‘reality’ are more important than others, thereby positing a salient interpretation or definition of a policy problem, the drivers behind it, and possible solutions, which can work in favour of a particular group [16]. Framing is, thus, key to policymaking as it informs understandings and perceptions of actors’ positions, legitimises their behaviour, and becomes a tool to challenge or reinforce power relations, with major implications for the success or failure of policies. Policymakers may use frames, deliberately or unconsciously, to construct a view of the world that is instrumental for them, allowing them to gain legitimacy and influence over the policymaking process [17]. A recent paper examining policy documents on AMR in Pakistan concluded that the framing used in these documents reflected relationships among various powerful groups of actors in the country and were likely designed to serve their interests in an attempt to influence the policymaking process [18]. Wernli and colleagues identified five predominant frames used in policy documents addressing AMR: AMR as a healthcare problem, as a development issue, as an innovation challenge, as a security issue, and as a One Health challenge [19]. They found that, although coherent, none of the frames alone was complex enough to describe and explain AMR, and who authored each document influenced the frames used. Policy innovation requires a clear understanding of the different frames used, how these frames reflect values and potential interests, and how they inform the priorities of the policymaking process for each epistemological community.

Although AMR is often framed as a One Health issue, with drivers encompassing human health, animal health, and environmental health domains, it is also reduced to individual responsibility, with behaviour change cited as a panacea for AMR [20]. Framing AMR as a lifestyle disease, i.e., a matter of individual choice to take or not to take antibiotics, is too easy and is not helpful in terms of dealing with the cogent issues.

AMR is framed and constructed in different ways by different groups with different motives [18,19]. These motives must be taken into account when looking at who is judged to be responsible for both driving and implementing solutions to AMR. The Social Construction Framework (SCF) [21] describes how people process information subjectively according to their internal biases, interests, beliefs, and values. The three key elements in the framework are (i) social constructions (perceptions of and symbols associated with different groups), (ii) target populations (who the policy is aimed at), and (iii) power relations. The SCF recognises that policy options are subjective, socially constructed, and related to how much power a group is perceived to hold. Understanding who is likely to influence AMR policymaking is key to constructing effective policy [22]. In this study, we used the SCF as a basis for our analysis, examining who is held responsible and why, but developed the idea further by recognising that AMR is multifaceted and that no true ‘target population’ exists, as everyone is a potential target.

Our aims were (i) to identify prevalent narratives and framing used by epistemological communities involved in the response to AMR in Tanzania (e.g., pharmacists, healthcare workers, veterinarians), (ii) to interrogate how this framing may inform policymaking, and (iii) to identify interventions that could be tailored to be effective for the different groups stated to be responsible for AMR.

## 2. Results

We interviewed 114 participants. Nine were members of the national Multi-Stakeholders Committee: one each from the Food and Agriculture Organisation (FAO) of the United Nations Country office, the Ministry of Livestock and Fisheries, and the Tanzania Medicines and Medical Devices Authority, and six from the Ministry of Health, Community Development, Gender, Elderly, and Children. We interviewed 25 laboratory technicians from public and private healthcare facilities, 23 health facility managers, 19 dispensers, 15 paravets, 12 livestock field officers, and eight pharmaceutical assistants. We also interviewed an Environmental officer from Ilala Municipality, a pharmacist from Kilosa District Hospital, and a staff member from an implementing partner.

First, we describe our findings in terms of who the interviewees framed as being responsible for driving the spread of AMR, including livestock farmers, pharmacies, individual consumers, medical professionals, local communities (groups of potential consumers), government, and donors. Second, we describe the actors who interviewees suggested could or should be responsible for addressing this spread.

### 2.1. Which Stakeholders Are Framed as Being Responsible for Driving AMR?

#### 2.1.1. Livestock Farmers

Interviewees stated that livestock farmers have little or no understanding of AMR; consequently, they do not consult veterinary specialists and instead self-treat their animals. They mentioned that farmers dispose of waste incorrectly and do not keep animal sheds clean. The predominant framing used was cost; farmers do not listen to veterinary specialists because they cannot afford the correct medicines. They use what they have always used, even if this is a medicine nominally for human use.

‘*When you go to provide services to a livestock keeper, let us say he called you to treat a certain disease which his livestock have, when you provide treatment he will always be there watching, he copies everything that you are doing, he will take that medicine and take a good look at it, they don’t know how to read but he will make sure he memorises it in his mind. So, next time he will do everything himself, so that gives us a challenge in solving the livestock problem, but we sensitise them many times that, if something happen, they should involve us.*’

[Paravet 1].

‘*In the community, people can use a certain drug without professional prescription, so they may use a certain medicine which in reality is not specific for a disease they intend to cure. So that creates drug resistance in livestock, and it becomes difficult to cure it […] livestock keepers should listen to livestock officers’ advice on the use of livestock products when their livestock uses antibiotics. Moreover, they should consult the livestock officer before using any antibiotic drug so as to reduce the problem of drug resistance and to get good results, rather than using many drugs at once without professional advice. That will create more problems and the government will incur costs to control that problem.*’

[Livestock Field Officer 1].

It was reported that farmers are often unable to afford to stop milking their animals or selling their meat to allow the antibiotic withdrawal period to elapse, which puts antibiotic residue into the human food chain. Livestock keepers also tend to do what they have always done and are sometimes not amenable to listening to advice from professionals.

‘*Concerning this problem of antimicrobial resistance as I said, since we are at a low level, we are the ones who know where the problem is. In Europe, this problem is not huge; for example, if chickens are suffering from Newcastle [disease], the government will take all the chicken and compensate a livestock keeper, but here we can’t do that. That is why it is difficult to tell a person to withdraw milk for 14 days when he depends on that milk for everything, or you can’t stop a person from selling meat just because his livestock is on antibiotic treatment and it is very difficult to follow up. This is where the problem of drug resistance in human beings started from.*’

[Paravet 2].

‘*[…] Maasai livestock keepers have traditional issues; you may go there with your professional idea but they don’t take it into consideration […] the medicines they use have no good quality; they buy medicines at the auction where they display medicines in the sun for almost 5 months but they take that medicine and use it on cows, which causes resistance and an increase in other unknown diseases, until they come to see you when the situation will be worse.*’

[Livestock Field Officer 2].

#### 2.1.2. Pharmacies

Some interviewees commented on pharmacies illegally selling antibiotics over the counter without a prescription. This is intertwined with the lack of enforcement of regulations, as pharmacists can be confident that they will not be penalised for these practices.

‘*One important thing in our pharmacies is we have policies, but they have not been enforced to prevent selling of drugs without a doctor’s prescription where the prescription is based on laboratory test results. Someone goes to the pharmacy and feels they have a UTI [urinary tract infection], so they buy antibiotics. So, there is a need to have over-the-counter prescriptions.*’

[AMR Programme Coordinator].

One dispenser stated that although in an ideal world doctors should always be consulted before antibiotics are dispensed, this does not happen in practice.

‘*In my opinion, we should keep on educating the community as we are doing. Moreover, you should help us to educate people who sell medicines in pharmacies that they should not give clients medicines without a doctor’s prescription […] we should control a system of selling medicines without a doctor’s prescription; that will help to control the problem of drug resistance […].*’

[Healthcare provider 1].

This quote illustrates the fact that people are making efforts to educate communities on the issues around AMR and what they can do to help prevent it spreading.

‘*Resistance happens because we have failed to control the sale of medicines in the street; most people who are selling in street pharmacies have poor medicine knowledge, so there should be control of medicines.*’

[Healthcare provider 2].

#### 2.1.3. Consumers

Many interviewees framed individual consumers as being partly responsible for the spread of AMR, for example, not finishing a course of antibiotics, but did not often clarify why they may behave in this way. A representative of the FAO, an agency of the United Nations, stated that individuals are responsible up to a point, but that the public needs to be educated to understand why they need to complete the course, as this does not necessarily make intuitive sense to some people, particularly those with little education. The next quote suggests that labelling needs to be made more effective and comprehensible to consumers.

‘*We use the term antibiotics, while the prescription form is written as amoxicillin or erythromycin. An ordinary citizen can’t understand that […] they should instruct all pharmaceutical industries and importers to add the word ANTIBIOTICS in capital letters (bolded) so that one knows what I was told when I was prescribed antibiotics and that I need to complete the dose.*’

[FAO representative].

Some interviewees framed AMR as being caused by a lack of individual responsibility, with patients not finishing the course of antibiotics.

‘*I can say that it is caused by use of medicines because, for all the time I have been here, I realised that most patients don’t finish the dose, so if a patient doesn’t finish a first stage dose and when they come back, you prescribe them another medicine, they may not finish that dose.*’

[Dispenser 4].

This is obviously linked to a lack of awareness and an understanding of the potential results of not finishing a course.

‘*You may find that a person may come to seek treatment here but after 2 days they come back. When you prescribe medicines for that client, they tell you that I have those medicines at home; when you ask them why they didn’t finish a dose, they tell you that, when they started feeling better, they stopped taking them. So, we have to educate them that, by doing that, it will cause drug resistance; if that happens, those medicines won’t be able to cure then anymore since they don’t finish a dose.*’

[Dispenser 8].

Furthermore, consumers who are living in poverty may have little choice but to buy one antibiotic tablet at a time and, thus, are unlikely to complete a prescribed course of drugs. They may not be able to afford the specific drugs prescribed for them; hence, pharmacies will dispense a different, possibly less targeted and effective, antibiotic.

‘*When patients take the responsibility of going to buy medicines at the pharmacy… if a doctor prescribed ampiclox, when that patient goes to the pharmacy, they say that the medicine cost is 3000 [Tanzanian shillings] but they have 1500, so they may ask a pharmacy seller for any other medicine that can help. They take another medicine which is at a low level compared with the medicine a doctor prescribed, which may also be an antibiotic but it may not be the right medicine for the disease that the patient has, which will cause resistance in the end.*’

[Healthcare provider 3].

‘*[…] a patient may tell you to prescribe half dose first and that they will come for the rest; this mainly happens in pharmacies. So, that patient takes half dose by promising they will come to buy another half dose, but, after finishing the first half dose and feeling better, they don’t go to buy another half dose, and, when they get sick again, they go back to the pharmacy and buy a half dose.*’

[Healthcare provider 16].

Consumer expectations are also an issue, as people know what worked for them the last time they were ill, so they ask for these antibiotics at the pharmacy without consulting a doctor. They need to be made aware that the antibiotic, although it worked once, will not necessarily work a second time, particularly for a different illness, and this may encourage resistance as it does not target the illness they are currently experiencing. This is framed partly as individual responsibility and partly as a need for better or more education.

‘*People have been used to the idea that, when they use a certain medicine they get better; maybe that person was prescribed a certain medicine and that medicine helped them, so the next time they get sick, they won’t go to see a doctor and will just go to buy that medicine and use it; so, they will keep taking the same medicine when they feel sick and, in the end, the disease becomes resistant. So, education should be provided in the community that they should not be taking medicines regularly […].*’

[Dispenser 13].

Consumers may also hear about effective drugs from word of mouth, but do not realise that these drugs need to target their specific illness; it is not one size fits all.

‘*However, sometimes, the problem is also patients; a patient may force a doctor to prescribe them certain medicines just because they were told that those medicines are effective.*’

[Environmental Officer].

One interviewee mentioned that, when issues around the correct selection of antibiotics are explained to patients, they do understand, and this is hopefully reflected in their behaviour, as they take the correct drug. This suggests that, in certain contexts, making consumers aware may work.

‘*That [patients requesting antibiotics] is one of the biggest challenges in treatment; clients may come with such suggestions, but we usually counsel them on which medicine will be appropriate according to the sickness they have, and I can say that most clients they understand when you explain it to them clearly.*’

[Healthcare provider 4].

#### 2.1.4. Medical Professionals

All dispensers interviewed stated that the community should be educated about AMR; none of the dispensers themselves received any specific training on AMR. Many of the health service provider interviewees, the majority of whom interact with the public on a daily basis, framed AMR as an issue of individual responsibility.

‘*I use the knowledge I acquired at college, for example, to tell a client the importance of taking medicines on time and the effects of taking medicines without a doctor’s prescription. I tell them that they should consult a doctor first before taking medicines so as to prevent antibiotic resistance.*’

[Dispenser 1].

‘*You may find that a patient is taking medicine for 3 days only; when they start feeling better, they stop taking medicine; so, after a certain period of time, they start getting sick again. Then, they come at the facility and say that they were prescribed medicine but started feeling sick again. If you ask them questions, you will realise that they didn’t finish a dose or you may find that they shared the medicines with a relative; so, we tell them that they should not do that.*’

[Healthcare provider 1].

If service providers are educated about AMR, interviewees suggested that they, in turn, will be able to inform their clients of the relevant issues.

‘*More education should be provided to service providers and to the community so that they can know how to use antibiotics effectively.*’

[Dispenser 1].

One interviewee framed AMR as being a result of misuse of antibiotics, but also stated that some doctors were complicit in the issue and overprescribed in an attempt to earn extra income.

‘*[…] a person is using medicines without following treatment regime, without knowing which medicines they should start with, or a person may be described antibiotics when they can use normal medicines; a patient may have a simple case but a doctor prescribes many and strong medicines just to earn income […].*’

[Healthcare provider 5].

Related to this is the issue of doctors prescribing different, possibly less effective drugs for patients with little money or those who do not have health insurance that would cover the cost of their medication.

‘*[…] for patients who don’t have health insurance, it is a challenge. Sometimes, a patient may tell a doctor to prescribe medicine of low cost since they have a small amount of money. So, you know for sure that this patient won’t afford to pay for ceftriaxone, so you just prescribe them amoxillin so that they can get relief.*’

[Healthcare provider 10].

Interviewees highlighted that doctors should be responsible for dispensing not just prescriptions but also advice to their patients. Doctors are widely respected in communities, which can mean that, when pharmacies have run out of specific drugs a doctor has prescribed, consumers are not willing to accept a substitution that would be equally effective. The same trust they have for doctors is not shown for pharmacists.

‘*When a patient comes, they should be educated, and doctors should listen to a patient’s previous medication treatment so that, if possible, a patient can be given a different medicine. Patients trust doctors so much, so when they come to the dispensary and you tell them that we don’t have this medicine and we will give you another medicine, some patients refuse. Sometimes, they go back to a doctor to show the medicines we gave them; so, they should be educated.*’

[Dispenser 11].

One interviewee was adamant that medicine has become a business rather than a vocation. This leads to irresponsible prescribing, whereby medical professionals can earn more income, and this in turn is fuelling the spread of AMR as patients are prescribed medications that are ineffective for their specific illness. This is compounded by the fact that doctors sometimes do not listen to what a patient is telling them; they are more interested in getting the next patient into the room and earning another consultation fee.

‘*[…] Nowadays, most of the doctors are doing business; now, a patient can go to a doctor’s office and start giving a doctor instructions on which medicines they want; sometimes a doctor won’t concentrate when a patient is talking as they are busy with other things. A patient may be telling a doctor which medicines they have used but a doctor doesn’t listen, so they will just attend to a patient quickly so that they can leave.*’

[Environmental Officer].

Public and private practice also complicates the issues. One doctor suggested that private medical practices pursue profit and are maybe less likely to follow guidelines, even when these exist.

‘*For government hospitals, I think there are positive results [with guidelines] but, for private hospitals, I think there won’t be positive results since they are business-oriented.*’

[Healthcare provider 5].

Participants mentioned that doctors should routinely run resistance tests, but these are expensive and individuals may not be able to afford them; hence, doctors have no option but to prescribe as they see fit. One interviewee suggested that the Ministry of Health needs to ensure that inexpensive testing options are available so that they are affordable by everyone.

‘*In my opinion, there should be a procedure of conducting tests on patients and to prescribe them appropriate medicines according to the bacteria they have rather than prescribing medicines regularly. Sometimes, patients may be given a certain medicine for UTI; when it fails, the doctor tries another medicine and, when that medicine fails, they try another medicine again. So, it will be better if we conduct a culture test so as to provide appropriate medication […] I think the ministry should improve this because medication touches people’s lives; so, they should consider reducing the cost for culture test since not all people can afford culture tests at 150,000 or 70,000 [Tanzanian Shillings].*’

[Healthcare provider 6].

Even when testing facilities do exist, doctors may not use these, which may be related to a lack of awareness of both the health professionals and the general public regarding the importance of testing to ensure that patients are prescribed an antibiotic that is going to be effective for them.

‘*Even in our big hospitals, people are being prescribed medication without any tests. So, we need to sensitise people to make sure that, for each drug we dispense, we take a sample for culture and sensitivity tests in order to either continue with the drug or substitute depending on the results.*’

[Ministry of Health official].

#### 2.1.5. Local Communities

It was suggested that local communities have not been involved in the design of the AMR national plan or invited to comment on its contents, and they have not been provided with awareness campaigns. Instead, the process was run by technocrats and health professionals with no input from service users at whom some of the NAP objectives are targeted.

‘*I’m not sure about the community involvement. In the sessions I attended, I did not see a participant specifically from the community.*’

[Ministry of Health official].

However, one interviewee mentioned that, now that the NAP is in place, certain specific communities are becoming more engaged and involved.

‘*The community from the grass roots, for example, livestock keepers or farmers, was not involved; it was difficult. Now, there is engagement because there are livestock keeper associations, so it is easy to get representatives; however, there were no such associations back then, so it was hard to get representatives.*’

[Ministry of Livestock and Fisheries representative].

#### 2.1.6. Government

Interviewees often stated that AMR policies prioritise human rather than animal or environmental health, and this was reflected in the fact that the human health sector was more involved in designing the NAP.

‘*[…] environmental people see that the environmental issue isn’t covered much although we included waste management. So, if you read the plan, the area which isn’t much covered is the environment.*’

[TMDA Medicines Coordinator].

‘*When you talk about these issues involving more than one sector, the human health sector has often taken a big part (okay); even in this exercise, it took a big part; even in the plan, it took a big part. However, at the end of the day, it has to touch other sectors because they provide a response to human health. Human health is leading because it is a priority.*’

[FAO representative].

There was a perceived lack of coordination among the different sectors, which remain in their silos and do not easily work together. This results in a duplication of effort.

‘*We have one health desk even though all the organisations have not been streamlined to work together. One of the efforts we have put in place is to have this MCC [Multisectoral Coordination Committee] session with some technical committees, but other sectors are not involved. You can’t blame anyone because that’s how our system has been from the beginning. Everyone is working alone and, at the end of the day, you get duplication of effort because of not working together. Even in terms of administration, each organisation has its own leader, which is very challenging.*’

[AMR Program Coordinator].

Participants emphasised that policies that do exist are not enforced effectively, which drives the spread of AMR. This lack of regulation may also mean that AMR is not accepted as a serious issue, as, if it was, the government would be addressing the problem. However, one interviewee, while agreeing that enforcement is an issue, argued that education needs to come first, as, if communities do not know what is required of them, they will not be able to comply.

‘*As I said, we should enact laws that will enforce people to do what is required. It is not an issue of like or not, it is a legal issue. However, people should not be forced by the law without being educated. Provision of education should come first; people should be told what is required and the consequences to make it easier to monitor.*’

[FAO representative].

Another interviewee stated that sufficient laws exist but that enforcement of these is lacking, and that it is the government’s responsibility to rectify this situation.

‘*Enforcement. If you look there are laws everywhere that are not followed. So, the government and responsible authorities overseeing this area should ensure enforcement is done.*’

[TMDA Medicines Coordinator].

One interviewee suggested that the government should enforce inspection of street medicine shops, which are currently unregulated, to try and ensure that consumers receive the medicines they have actually been prescribed by a medical professional.

‘*When you talk about the hospital, it means guidelines, but do people who sell medicines in the street use guidelines? When a patient comes here, we give them medicines according to the guideline, but, after some time, that patient suffers from the same problem, then you realise that they were given another medicine at the street medicine shop. If the government doesn’t have a system of conducting inspection of medicine shops to see if they follow procedures in dispensing medicines, this problem won’t end […] The government should form a team to investigate if people are given proper medication, to investigate if pharmacies dispense medicines by considering the doctor’s prescription.*’

[Healthcare provider 2].

Lack of enforcement of policies was also noted in animal health. The next quote illustrates some of the challenges described by our participants. In this case, the interviewee explained that, similarly to the human health sector, policies that do exist around antibiotic sales to livestock farmers are not enforced or implemented. They suggest that the government should clarify who is able to sell antibiotics, and where they can sell these.

‘*I think there are those policies at the livestock department even though they are not implemented because there is a free market for medicines; a Maasai sells medicines to his fellow Maasai, so there is a policy, but the implementation is weak. For example, when you go to the livestock auction and tell people to stop selling medicines unless they have a licence, they will tell you that, at other auctions, people sell medicine so you can’t control them. So, there are policies but there is no policy management; however, if the government would release a statement that a livestock keeper is not permitted to sell certain medicines including antibiotics and vaccines, things would be better.*’

[Livestock Field Officer 3].

#### 2.1.7. Donors

There was a perceived lack of donor funding and lack of coordination among the different sectors. One interviewee commented that the AMR agenda is donor-driven, and, when organisations leave a country, the situation becomes complicated, especially if there is not enough domestic funding to continue programmes.

‘*We are supposed to know all stakeholders who participate in AMR, but we don’t and we have to know them for mapping. Another challenge is that, for the environment, it is not well coordinated and needs extra effort. Another challenge is insufficient funds compared to existing activities. If I look clearly, I see that this AMR is more of a donor-funded project than a government-funded one, so when donors leave, the situation will be difficult.*’

[TMDA Medicines Coordinator].

However, another interviewee stated that the Ministry of Health is cognisant of issues around financial support from donors, and a budget now exists to facilitate plan implementation.

‘*The main challenge which faces many areas is a lack of funding, because plan implementation needs money in one way or another; most of the time, we depend on donor support, but we are thankful that now our government has given the health ministry priority in terms of budget.*’

[Healthcare provider 22].

### 2.2. Who Is Framed as Having the Ability to Tackle AMR?

The Tanzanian Ministry of Health was described by most interviewees as the pivotal stakeholder in the development of a response to AMR, which is consistent with the framing of AMR as predominantly a human health issue that should drive policymaking. Other actors mentioned included the FAO, WHO, and TMDA.

Three interviewees commented that political will is present and that the Ministry of Health is an effective policymaker, having designed a budget to accommodate recommendations from the NAP without relying on external funds from donors.

‘*The success that I currently see is political will; the government has taken this seriously. However, the issues of governance are also why we now have had MCC sessions to coordinate everything that is happening in the country regarding antimicrobial resistance. This is good because things are run in order and not arbitrary. Moreover, a government commitment is needed, where the government does not rely solely on donations from donors like FAO, WHO, and others. However, in this session, we also said that they should allocate a budget in their annual plans. It is the government’s commitment that, even if these projects do not exist, the AMR will continue to be implemented.*’

[FAO representative].

Another interviewee commented that political will exists in Tanzania, and this is crucial for communities to listen and adopt practices that are designed to help control AMR.

‘*One of the successes is that there is political will; you know, in all health programs, you must first get political will or political commitment. So when, you get political commitment, that is a very big success because high-level leaders are the ones who plan national strategies; they have national vision, and they take that matter as a priority. So, they may even allocate a budget, and a high-level leader may talk about it. In involving the community, when I go to talk with the community, they may not listen, but, when a member of parliament or the ministry goes to talk to people, they will listen; that is a big advantage of political will.*’

[Healthcare provider 22].

The government is perceived as an active partner in the response to AMR, with one interviewee commenting that the Ministry of Health in particular has been at the forefront of the response.

‘*However, in general, the health ministry had a very big contribution because they were involved in situation analysis to see what is missing and what is not giving good results for some infections. So, we gathered a lot of information from that sector.*’

[Ministry of Livestock and Fisheries representative].

A second interviewee echoed the key role of the Ministry of Health, which is leading the response to AMR in Tanzania.

‘*In reality, this issue is managed by the health ministry; its source is the health ministry, people think that the issue of antimicrobial resistance concerns human beings only, the health ministry is the one managing human health, the leader of the central committee comes from the health ministry, and the secretariat is formed by many people from the health ministry; therefore, since we said it is a one health concept, the health ministry must take leadership responsibility to incorporate people from other sectors like wild animals into this issue.*’

[Healthcare provider 22].

One interviewee stated that the government is effectively educating the general public on the risks around AMR and taking antibiotics in particular.

‘*So, we educate them that it is not a good thing to take medicines without testing. Moreover, sometimes cars pass in street to announce that not all fevers are malaria fevers. The government is doing a good job in that area; people are being told that they should not use medicines without testing, and we tell them that antibiotics are very, very strong.*’

[Laboratory 4].

The FAO presents itself as having an advisory role and potentially providing various types of support to the government.

‘*The FAO is there for providing support. We, therefore, advise the country about things which are happening and things that will happen. We are ready to provide support in terms of technical and financial.*’

[FAO representative].

The WHO was also mentioned as a key actor in the efforts to prevent the spread of AMR, working in conjunction with the Ministry of Health and to an extent the Ministry of Agriculture.

‘*We had a big push in the Ministry of Health although all ministries were fully involved because our main stakeholder is the WHO and we are part of it. WHO brings us policies, but it is up to us to adapt them. Alternatively, they bring you different models through the Ministry of Health. So, the Ministry of Health was mobilised before mobilising other sectors although the ministry of livestock and agriculture had its own guidelines.*’

[AMR Program Coordinator].

Other donors and governmental bodies were mentioned as key stakeholders in the NAP process by some interviewees.

‘*The main stakeholders are, since the plan is about one health, ministries related to human health and veterinary health and stakeholders involved in agriculture in general, as well as various research institutions and professional institutions, such as SUA and MUHAS, and development partners, such as the Global Development Organisation, Food and Agriculture Organisation, and other stakeholders like the CDC.*’

[FAO representative].

The role of international bodies and concomitant financial support for meeting attendance was discussed by one interviewee.

‘*International institutions were also part of the plan preparation; for example, we had representatives from the FAO, WHO, OIE, and the World Animal Organisation, while there were also interested parties like our partners from the American Society of Microbiology. Moreover, I remember CDC were also there; they also facilitated and they supported the meetings because participants had to travel, and I remember one of the sessions was conducted in Morogoro, so people travelled from Dar es Salaam to Morogoro; so, they were supporting us financially.*’

[Ministry of Livestock and Fisheries representative].

These national and international bodies were generally perceived as supportive of efforts around AMR, with very few critiques of the governmental or donor positions.

## 3. Discussion

In this study, we identified how different actors were framed by interviewees with regard to their involvement in the response to AMR in Tanzania, while recognising that those groups or actors that potentially influence policymaking are not necessarily coherent epistemological communities. Our analysis suggests that the drivers of AMR in Tanzania are multifaceted and complicated, and that no single ‘target population’ is believed to be responsible for the spread of AMR in the country. Livestock farmers, pharmacies, medical professionals, consumers in local communities, and the government were all mentioned as bearing at least some of the responsibility for AMR. Human health is strongly emphasised in policies and interventions, with a concomitant neglect of potential animal health and environmental health drivers. This finding is replicated in other countries, for example, Pakistan [3]. This emphasis on human health is likely to take resources, both human and financial, away from the animal and environmental health sectors and focus political will on human health, which is perceived as easier to address. Actions aimed at human health may also be more visible and result in public approval of governmental policies. Interestingly, many interviewees commented that political will is evinced by the Tanzanian government, which appears keen to implement effective policymaking [14]. This is possibly a surprising finding, as governmental support, although key to designing and implementing effective policies to address AMR, is not often visible. Interestingly, a few interviewees discussed the underlying lack of public health provision and infrastructure in Tanzania, although the lack of sufficient laboratory diagnostic facilities was mentioned as a driver of the spread of AMR, as this prevents a definite diagnosis and, thus, correct antibiotic prescription.

Stakeholders, drivers identified, and possible solutions are presented in Table 1.

### 3.1. How Does This Framing Inform Policymaking?

The most frequently discussed aspect of drivers of AMR in our interviews was education and lack of awareness, both in communities and in animal and human health professionals. The general public was framed by some interviewees as being unaware of the importance of, for example, finishing antibiotic courses, while others suggested that people do understand the significance of their actions but are either uninterested in the outcome of these or are unable to take them into consideration—for example, the livestock farmer who ignores the antibiotic withdrawal period as they cannot afford not to sell the animal’s meat or milk. This is related to the individual responsibility discourse, which frames individual behaviour change as the key solution to the spread of AMR [20]. This is facile; it is too easy to frame taking or not taking antibiotics, finishing a course of antibiotics, or not respecting a withdrawal period as individual responsibility, without taking into account the multitude of other factors that drive behaviour. In a country like Tanzania, with many other complicating issues such as antibiotic sales without a prescription [7] and a lack of effective regulation of antibiotic use in humans and animals [8,9,10,11], this is particularly unhelpful. It should be kept in mind that communities were perceived as having played no part in the design of the AMR NAP. There was a general consensus among interviewees that awareness is essential, although this does not necessarily translate into effective behaviour change. A further issue is that it would be difficult to target an education or awareness campaign at communities that may have very different backgrounds and understandings.

Although the Tanzanian Ministry of Health was widely perceived by interviewees as playing the lead role in the formulation of the AMR NAP, international organisations were framed as playing a significant role in the response to AMR in Tanzania. Funding from international organisations is perceived as essential for any kind of response to issues that should be addressed by national governments. One interviewee, whose job role was focused on animal health, stated that human health is a priority. FAO provides financial support for the implementation of NAPs and, therefore, has a voice in the process. Another interviewee stated that the JEE findings, suggesting that Tanzania is not equipped to deal effectively with AMR, were a strong catalyst for starting work on the plan. This implies that indirect pressure has come from the international community. Similarly, donor funding was seen by some interviewees as key to the successful implementation of any plan; thus, if donors exit a country or withdraw funding, a huge financial resource disappears, which will make implementation even more difficult. A 2020 report from the World Bank suggested that Tanzania’s spend on health is insufficient to allow everyone to access quality health services, and that per capita spend on health was 28.5 USD in 2017, an increase from 23.6 USD in 2010 [23]. The report also found that the health sector is over-reliant on donors, with the government providing approximately 40% of the total spend on public health [24]. A more sustainable approach may be to use external consultants to inform local efforts rather than being reliant on donors, who have their own interests and agendas. However, some interviewees stated that there is a lot of political will from the Tanzanian government to work toward addressing drivers of AMR in the country, and that this will may help to an extent if donors stop funding projects.

### 3.2. What Interventions May Be Effective When Targeted at Different Groups?

It is easier to target policies at groups who are viewed negatively and judged to be less deserving of support (e.g., livestock farmers who ignore, for whatever reason, withdrawal periods) rather than those who are generally positively viewed (e.g., doctors and medical professionals). In addition, positively viewed groups have more power and influence and may be able to deflect policies that are aimed at addressing their behaviours. This becomes a vicious cycle, with positively viewed groups becoming more and more powerful and more and more able to influence policymaking, supporting legislation that benefits them and shooting down policy that does not. It then becomes much easier for authorities to take the line of least resistance and target policies and interventions at weaker groups, for example, consumers buying one or two antibiotics at a time. If the pharmacy selling them these drugs is closed down as part of a programme of reform, poorer consumers are the people who will suffer. Doctors are much less likely to be perceived as driven by personal motivation than they are as people who care for others and have their best interests at heart.

Targeting policies at different groups, rather than a simplistic one-size-fits-all approach, may help to promote robust and effective policies that will increase the likelihood of success. Interventions must be realistic and tailored to the context in which they will be implemented. The presence of political will and a respected and responsible government in Tanzania, mentioned by some interviewees, will be key to successful policy implementation, but this must also come with a financial commitment that is viable and does not rely too much on donors. Collaborations among stakeholders representing different One Health areas (human, animal, and environmental health) are going to be the cornerstone of any effective policy designed to address the drivers of AMR in Tanzania. This is a complex area; professionals are often siloed in their specialties and are reluctant to work with others. A lack of consistent funding, while this may not be so much of an issue in Tanzania, must also be taken into account. As discussed by most of the interviewees, the lack of awareness of and education around AMR has to be addressed. The general public will not change their behaviour unless they understand why doing so is essential, individually and at the country level. Tackling AMR should be framed in a way that makes benefits to the individual obvious.

Implementing, enforcing, and monitoring initiatives are also key to addressing AMR. Pharmacists who sell antibiotics over the counter without a prescription, doctors who prescribe without a laboratory report, and livestock owners who diagnose and treat their cattle themselves must all be tackled. Context-specific monitoring and enforcement must be put into place, and gaps and loopholes must be identified. For example, if a system was initiated to allow prescriptions to be sent directly from a medical practice to a pharmacy, there would be little room for dispensing error or amendment of prescriptions by the consumer. Livestock farmers who forgo selling meat and milk until the antibiotic withdrawal period has elapsed could be reimbursed by the government. A mixture of education, awareness, and enforcement should be implemented to encourage everyone to work together to slow down the spread of AMR in Tanzania.

### 3.3. Limitations of the Study

This was a qualitative study examining the perceptions of drivers of AMR in Tanzania and, thus, we did not focus primarily on solutions to all the issues that were identified and discussed in the interviews. We interviewed 114 people but recognise that their views may not have been representative of their colleagues and other people involved in the same field. This is a limitation common to all qualitative research.

## 4. Materials and Methods

### 4.1. Study Setting

This qualitative study was conducted in three districts in Tanzania in January and February 2020. Ilala district was selected because it is a densely populated urban area with a mixture of housing, agriculture, and industry, and it is heavily polluted with effluent from abattoirs, domestic wastewater, and septic tanks and pit latrines [25,26]. The population of Kibaha district is involved in large- and small-scale poultry and fish farming, which may involve the use of antimicrobials [27,28]. Kilosa district has a large population of pastoralists keeping cattle, sheep, and goats. This group is likely to self-treat their animals using antimicrobials [29].

We used a purposive sampling strategy to identify key informants from national government and health facilities in these districts. National policymakers were purposively selected from the ministries responsible for Public Health, Livestock and Fisheries, and Food and Agriculture. Other key informants were selected from the national regulatory authorities and agencies. These key informants were selected because of their role in the preparation and implementation of the NAP for AMR. At the facility and community levels, we interviewed key informants including laboratory technicians, livestock officers, paravets, healthcare facility managers, and pharmaceutical assistants and dispensers.

### 4.2. Data Collection

Two supervisors conducted a mapping exercise by visiting all three districts to identify a list of potential key informants under the guidance of the district officials. Based on the list of key informants generated from the mapping exercise and under the guidance of officials from the district and ward/village levels, we conducted in-depth interviews with key informants from the health, environment, and livestock and fishing sectors from national, district, and lower (ward and village/street) levels. All participants gave informed consent to be interviewed.

Face-to-face interviews were conducted in Kiswahili for 45 to 90 min. They were run by 12 research assistants with undergraduate-level training in qualitative research and coordinated by two researchers with postgraduate-level training in qualitative studies. The researchers had no existing relationship with the interviewees. A topic guide was used in the interviews, and interviewees were asked at the end of their interview whether they had any further comments or whether they wanted to discuss anything that had not been mentioned. All interviewees gave permission to record the interviews. Participants were able to choose the location of the interview, which usually took place at their workplace. The researchers took brief notes during the interview, and these were written up immediately after each interview.

### 4.3. Data Management and Analysis

All interview transcripts were transcribed verbatim and translated from Kiswahili into English. Some transcripts were back-translated to check that there was no loss of the original meaning. We used a thematic data analysis approach, which applies both inductive and deductive reasoning. A.D.B. and A.S.J. collectively identified and validated themes across a sample of transcripts, reaching thematic saturation when no new themes were apparent in the interviews [24]. A.D.B. and A.S.J. then conducted a line-by-line analysis on all interview transcripts, using NVivo 12 qualitative data analysis software (QSR International Pty Ltd., Melbourne, Australia, Version 12, 2018).

## 5. Conclusions

Our results suggest that many different groups help drive the spread of AMR in Tanzania and need to be involved for any response to be effective. However, this is not yet the case in Tanzania. Human health is currently perceived as driving the response, while other domains lag behind in their efforts. Likewise, for AMR programmes and NAPs to be successful, all sectors need to be involved, including civil society groups, community representatives, and those working in communities (e.g., primary care physicians). Yet, the NAP and current programmes largely fail to include these viewpoints. The perceived presence of political will in Tanzania is a significant step toward such a response. Any strategies to tackle AMR need to be tailored to the context-specific realities, taking into account constraints, beliefs, and power dynamics within countries.

## Figures and Tables

**Table 1:** Stakeholders, drivers, and solutions.

Stakeholder	Driver	Potential Solution
Livestock farmers	Lack of awareness of AMR.	Enhance education programme; enforce relevant legislation on sales of antimicrobials to farmers; reimbursement for farmers who lose money as a result of the antibiotic withdrawal period.
Pharmacies	Selling antibiotics over the counter without a prescription.	Enforce inspections of pharmacy prescription records; match with those from doctors.
Consumers	Expectations of which antibiotic should be prescribed.	Improve awareness of AMR, i.e., why using the correct antibiotic is important.
Medical professionals	Wrongly prescribing as tests are expensive.	Use inexpensive testing options.
Local communities	Lack of engagement.	Engage in planning/implementation.
Government	Lack of coordination among human, animal, and environmental health sectors.	Engage all sectors in planning and implementation of policies.
Donors	Lack of programme longevity.	Increase government budget for public health.

## Data Availability

The dataset analysed during the current study is available from the corresponding author on reasonable request.
